# Supplementary material for: Platform for isolation and characterization of SARS-CoV-2 variants enables rapid characterization of Omicron in Australia
Source: Nat Microbiol. 2022 May 30;7(6):896–908. doi: 10.1038/s41564-022-01135-7 (PMC9159941; doi:10.1038/s41564-022-01135-7)
Supplement: Supplementary file 1 — Supplementary Figs. 1–5. [file 41564_2022_1135_MOESM1_ESM.pdf]

nature portfolio

https://doi.org/10.1038/s41564-022-01135-7

Supplementary information

Platform for isolation and characterization of SARS-CoV-2 variants enables rapid characterization of Omicron in Australia

In the format provided by the authors and unedited

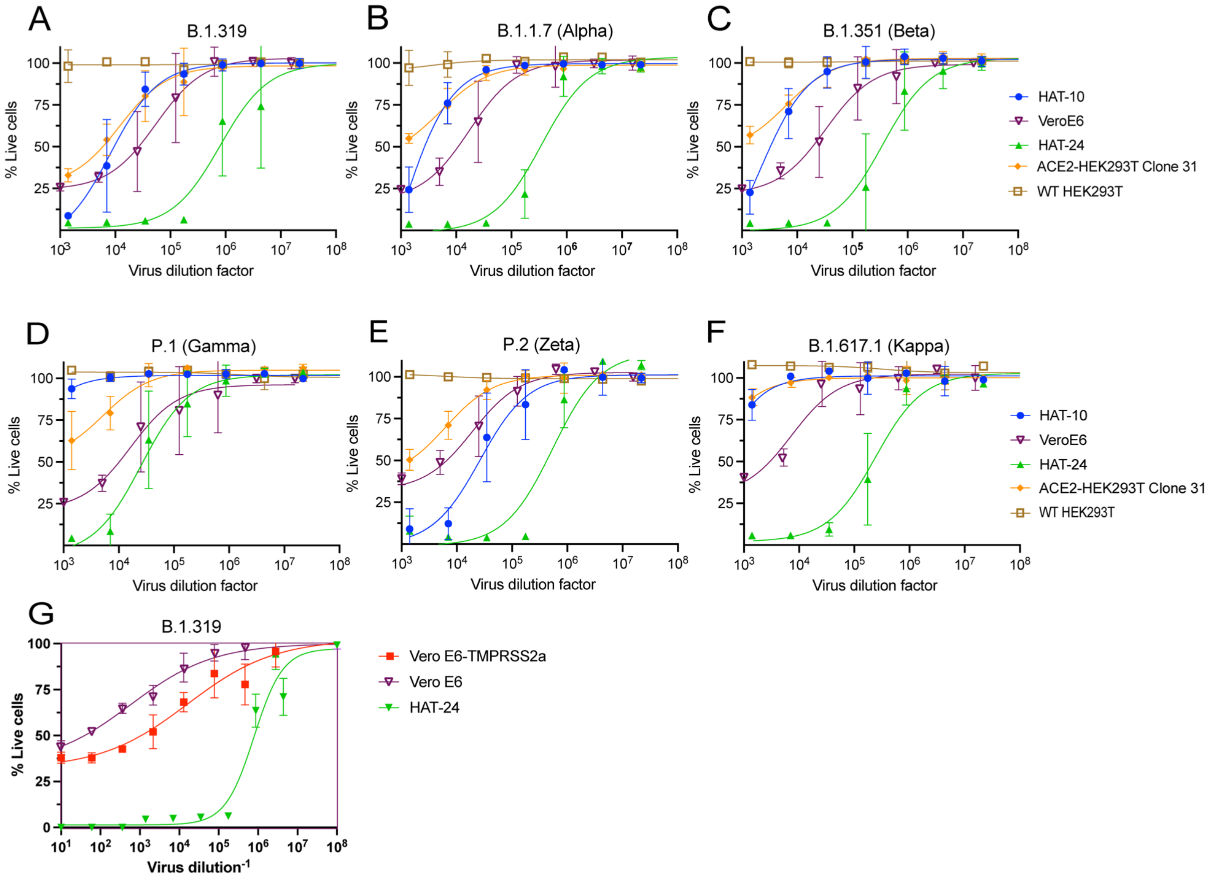

Supplement Figure 1. Relative sensitivity of cellular clones to SARS-CoV-2 CPE.

Each cell indicated was infected with **A.** early 2020 B1 clade B.1.319 **B.** Alpha **C.** Beta **D.** Gamma **E.** Zeta and **F.** Kappa to observe relative sensitivities to viral induced cytopathic effects. CPE was quantified 3 days post infection by staining the remaining nuclei with the live nuclei stain Hoechst3342/NucBlue. G. Represents an additional control using the Clade B variant B.1.319 to observe sustained CPE over three days in the VeroE6, Vero-E6-TMPRSS2 and HAT-24 lines. All standard deviations and mean values are presented from 4 technical replicates per dilution. Clone 31 is a Hek293T clone engineered to express ACE2 only. HAT-10 is a Hek 293T clone engineered to express ACE2 and TMPRSS2. Clone 10 was one of 23 clones that were observed to have an equivalent phenotype. HAT-24 was the only clone out of 24 to be observed to be highly permissive to SARS-CoV-2 infection and sustained CPE within 8 hours post infection. Parental Hek 293T cells and VeroE6 are presented are a comparison. Data is representative of > n = 10 independent experiments.

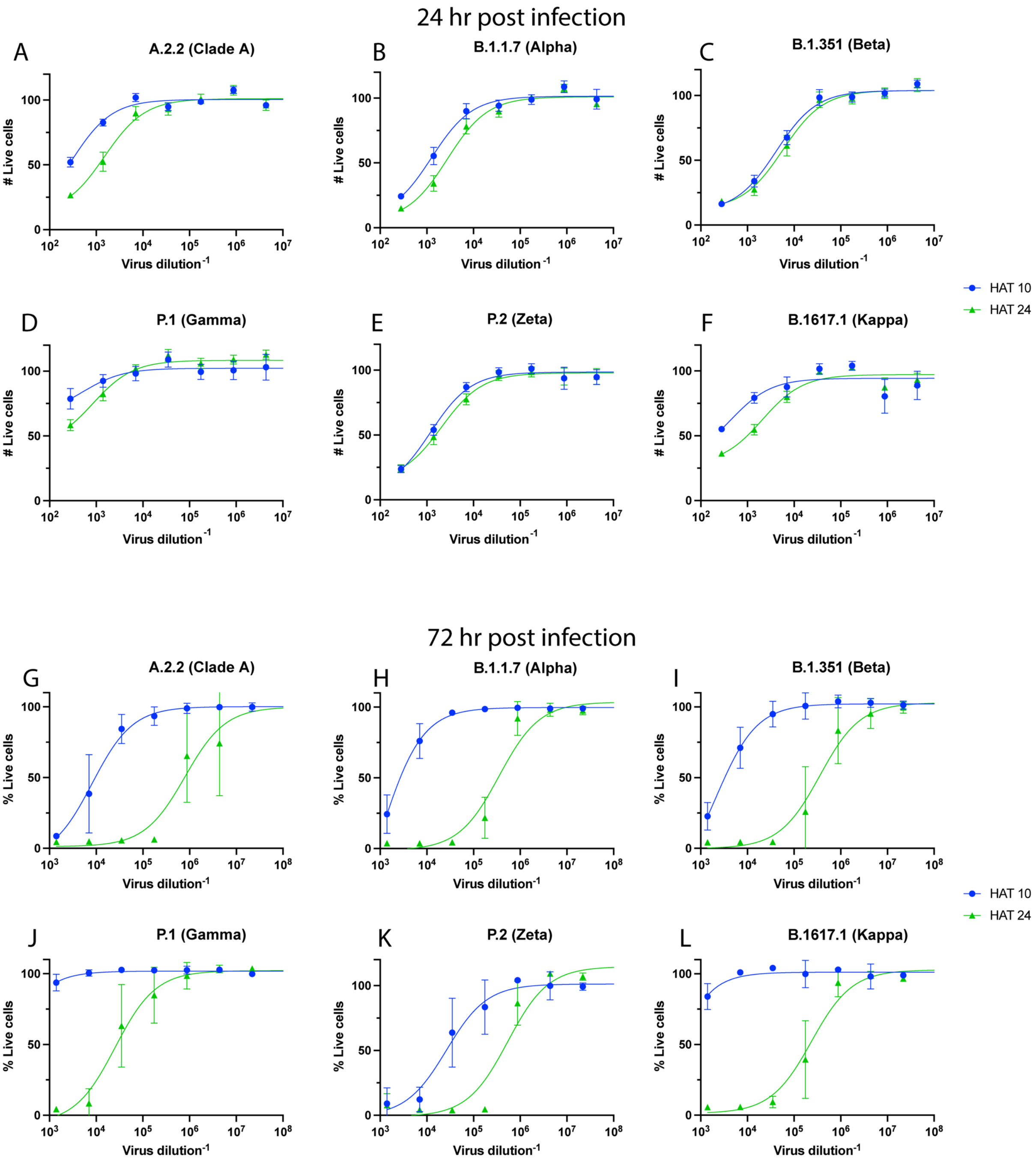

Supplement Figure 2. Relative sensitivity of cellular clones to SARS-CoV-2 CPE over time.
Each cell indicated was infected with **A. & G** early 2020 Clade A. A2.2 **B. & H.** Alpha **C. & I.** Beta **D. & J.** Gamma **E. & K.** Zeta and **F. & L.** Kappa to observe relative sensitivities to viral induced cytopathic effects. CPE was quantified either 1 day (**A-F**) or or 3 days (**G-L**) post infection by staining the remaining nuclei with the live nuclei stain Hoechst3342/NucBlue. Data is representative of > n = 3 independent experiments. All standard deviations and mean values are presented from 4 technical replicates per dilution.

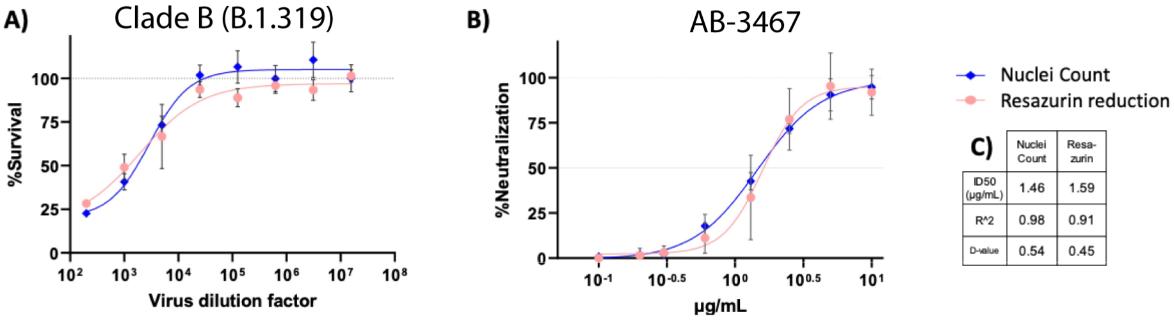

	Nuclei Count	Resa-zurin
ID50 (µg/mL)	1.46	1.59
R^2	0.98	0.91
D-value	0.54	0.45

Supplement Figure 3: Alternate low cost readout for rapid SARS-CoV-2 live virus neutralization assay.

A. Viral titration and B. neutralization assays using the human monoclonal 3467 were validated for an alternative readout method using metabolic reduction of resazurin to simplify the high content read out to a standard optical plate reader. HAT-24 cells were either pre-stained before seeding with Hoechst3342 nuclear dye for endpoint readout via high-content microscopy and automated whole-well nuclei counts (blue curve) or seeded unstained for endpoint readout via 3h resazurin reduction and whole-well fluorescence measurement (pink curve). Cells were seeded in 96-well plates at 6.4 x 10^4 cells/well and received an equal volume of B.1319 virus in increasing concentrations (A), or a fix amount of virus previously incubated for 1h with increasing concentrations of the monoclonal antibody mAb3467 (B). Plates were incubated for 20h at 37°C until endpoint readout, and percentage of survival was calculated relative to average signal from uninfected controls, with percentage of neutralization calculated as described in the methods section. **C)** 50% inhibitory dose (ID50) for mAb3467 and the dose.

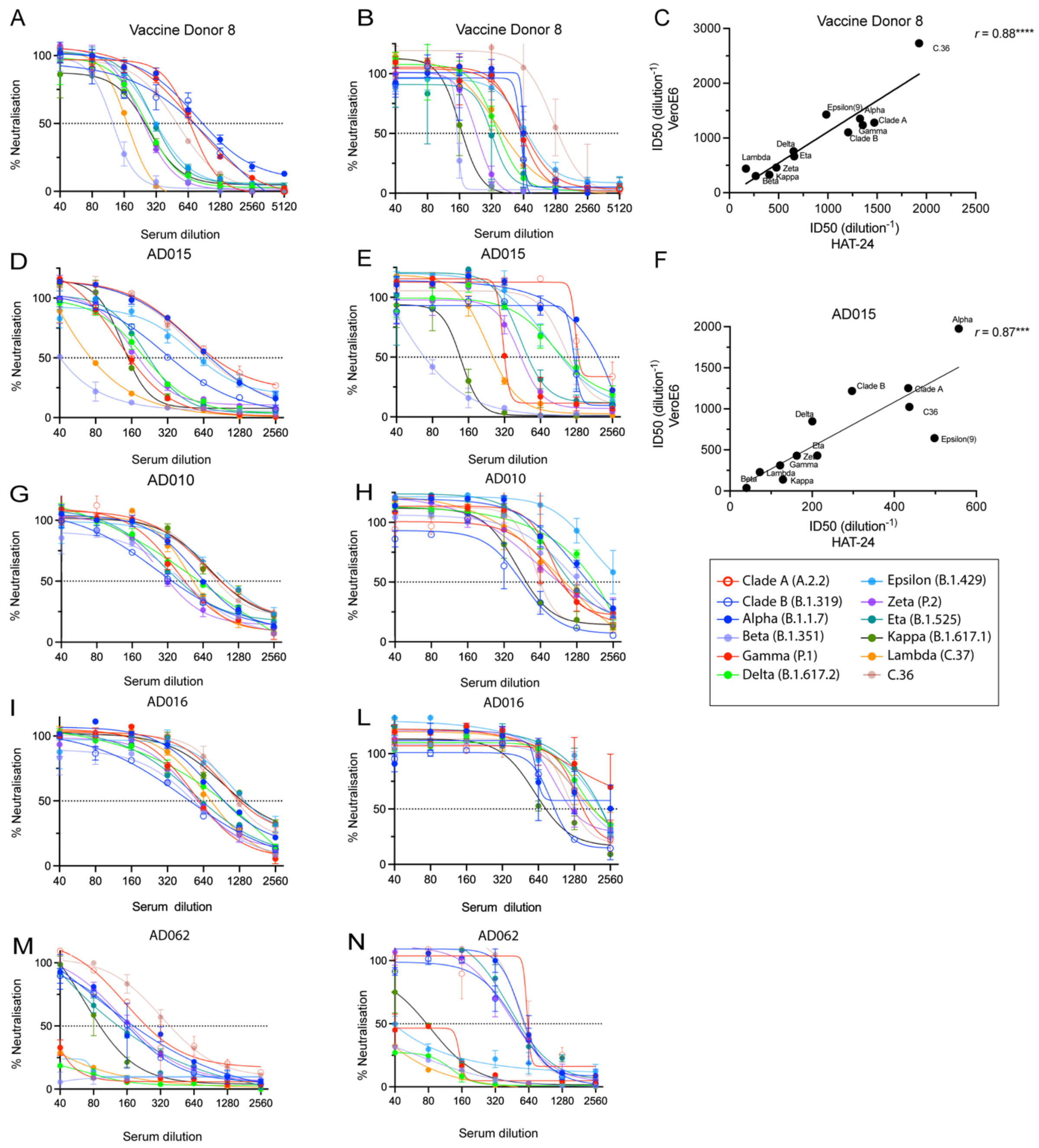

<u>Supplement Figure 4: Representative breath of donor specific responses for 12 primary variants.</u>

Representative BNT162b2 donor in **A.** HAT-24 R-20 format versus **B.** VeroE6. **C.** Correlation between HAT-24 and VeroE6 IC50 values from A & B. **D-F** Representative convalescent donor with an eroding response to contemporary SARS-CoV-2. **D.** HAT-24 R-20 format versus **E.** VeroE6 **F.** with respective IC50 value correlations for D & E. **G-L** neutralisation responses in two convalescent donors with good breadth across all variants. **G& I** are in HAT-24 and **H & L** in VeroE6. **M & N.** Donor with initial good response to early clades but lack of end point titers to Gamma, Beta and variants with the spike L454R or L452Q (Delta, Kappa, Lambda and Epsilon).

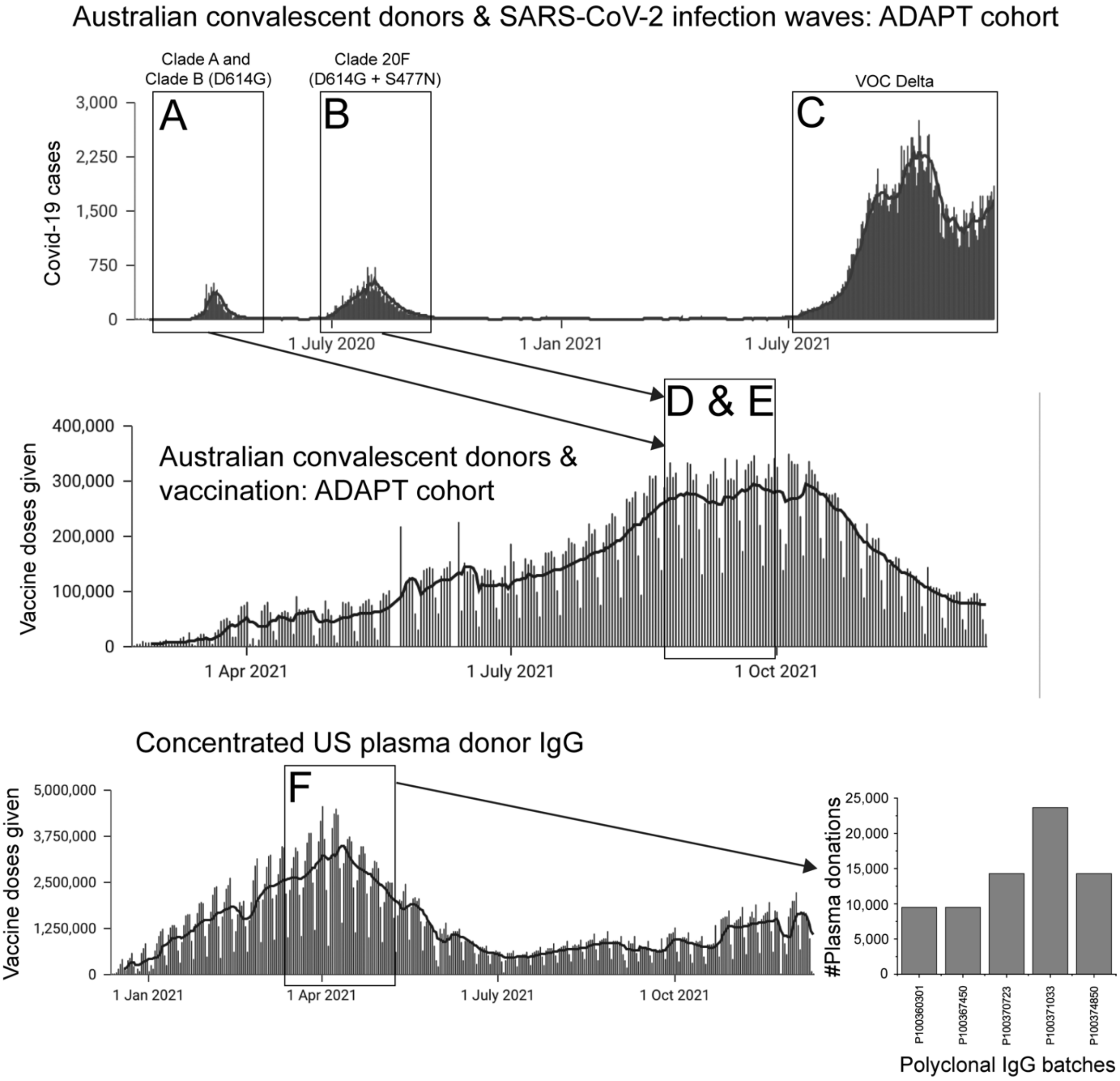

<u>Supplement Figure 5: Source of high titre serology samples and polyclonal IgG for Omicron characterisation obtained during the COVID-19 pandemic.</u>

Upper panel **(A-C)**: The ADAPT cohort and a summary of COVID-19 incidence over time in Australia. In brief, ADAPT is a community cohort of approximately 200 patients. The samples studied were from those with the highest neutralisation capacities to the ancestral strain. Herein we present the timeline of the COVID-19 pandemic in Australia illustrating the national total number of daily COVID-19 cases during each wave of infection. **(A-C)** Convalescent serum donors from the Australian ADAPT cohort collected from **(A)** Clade A and Clade B (D614G) (first wave) (n=10), **(B)** Clade 20F (D614G + S477N) (second wave) (n=10) and **(C)** the third (VOC Delta) wave (n=10). **(D-E)** Double dose vaccinated serum donors from the Australian ADAPT cohort collected during convalescence from **(D)** Clade A and Clade B (D614G) (first wave) (n=10) and **(E)** Clade 20F (D614G + S477N) (second wave) (n=9). Vaccinated donors are split equally between ChAdOx1 nCoV-19 and BNT162b2 and represent the peak responses following vaccination (one month post-second dose). **(F)** Concentrated human hyperimmune IgG from US plasma donors collected between March and May 2021 (left panel) including the number of plasma donations per polyclonal IgG batch tested (right panel). Not shown is the convalescent only polyclonal IgG, which was derived from US convalescent donors over the period of September and October 2020. This polyclonal IgG constituted approximately 5000 plasma donations over this period. (A-E) are derived from data courtesy of https://ourworldindata.org/.
