# Supplementary material for: Platform for isolation and characterization of SARS-CoV-2 variants enables rapid characterization of Omicron in Australia
Source: Nat Microbiol. 2022 May 30;7(6):896–908. doi: 10.1038/s41564-022-01135-7 (PMC9159941; doi:10.1038/s41564-022-01135-7)
Supplement: Supplementary file 2 — Reporting Summary [file 41564_2022_1135_MOESM2_ESM.pdf]

nature portfolio

Corresponding author(s): Stuart Turville

Last updated by author(s): Apr 7, 2022

Reporting Summary

Nature Portfolio wishes to improve the reproducibility of the work that we publish. This form provides structure for consistency and transparency in reporting. For further information on Nature Portfolio policies, see our Editorial Policies and the Editorial Policy Checklist.

Statistics

For all statistical analyses, confirm that the following items are present in the figure legend, table legend, main text, or Methods section.

n/a	Confirmed	
☐	☒	The exact sample size (*n*) for each experimental group/condition, given as a discrete number and unit of measurement
☐	☒	A statement on whether measurements were taken from distinct samples or whether the same sample was measured repeatedly
☐	☒	The statistical test(s) used AND whether they are one- or two-sided<br>*Only common tests should be described solely by name; describe more complex techniques in the Methods section.*
☒	☐	A description of all covariates tested
☐	☒	A description of any assumptions or corrections, such as tests of normality and adjustment for multiple comparisons
☐	☒	A full description of the statistical parameters including central tendency (e.g. means) or other basic estimates (e.g. regression coefficient) AND variation (e.g. standard deviation) or associated estimates of uncertainty (e.g. confidence intervals)
☐	☒	For null hypothesis testing, the test statistic (e.g. *F*, *t*, *r*) with confidence intervals, effect sizes, degrees of freedom and *P* value noted<br>*Give P values as exact values whenever suitable.*
☒	☐	For Bayesian analysis, information on the choice of priors and Markov chain Monte Carlo settings
☒	☐	For hierarchical and complex designs, identification of the appropriate level for tests and full reporting of outcomes
☐	☒	Estimates of effect sizes (e.g. Cohen's *d*, Pearson's *r*), indicating how they were calculated

Our web collection on statistics for biologists contains articles on many of the points above.

Software and code

Policy information about availability of computer code

Data collection	InCell Analyzer HS2500 (Cytiva) v7.3-17022
Data analysis	GraphPad Prism ver 9.3.0; InCarta (Cytiva) v1.12.3782849

For manuscripts utilizing custom algorithms or software that are central to the research but not yet described in published literature, software must be made available to editors and reviewers. We strongly encourage code deposition in a community repository (e.g. GitHub). See the Nature Portfolio guidelines for submitting code & software for further information.

Data

Policy information about availability of data

All manuscripts must include a data availability statement. This statement should provide the following information, where applicable:

- Accession codes, unique identifiers, or web links for publicly available datasets
- A description of any restrictions on data availability
- For clinical datasets or third party data, please ensure that the statement adheres to our policy

Whole-genome viral sequences for the SARS-CoV-2 variants used in this study have been deposited to GISAID and are publicly available as of the date of publication. Accession numbers are listed in Table S10. IC50 or cut-off titres values for all cohorts listed herein are in Tables S1-9. Source data for figures is available at: https://doi.org/10.6084/m9.figshare.19530550.v1Source data for supplementary data is available at: https://doi.org/10.6084/m9.figshare.19523401.v1

Field-specific reporting

Please select the one below that is the best fit for your research. If you are not sure, read the appropriate sections before making your selection.

☒ Life sciences ☐ Behavioural & social sciences ☐ Ecological, evolutionary & environmental sciences

For a reference copy of the document with all sections, see nature.com/documents/nr-reporting-summary-flat.pdf

Life sciences study design

All studies must disclose on these points even when the disclosure is negative.

Sample size	Of the >200 ADAPT participants, a panel of 25 representative donors was chosen based on serum neutralization titres against 'widtype' B.1 (B.1.319)-clade virus (4 donors each for endpoint titres of: ≤40, 40, 80, 160, 320 and ≥640). A second cohort was composed of 24 healthy adult vaccine-recipients, who received the BNT162b2 vaccine in 2021. Serum samples were collected three weeks post second-dose vaccination. In the convalescent cohort (from ADAPT) only donors that showed positive serum neutralization titres against 'widtype' B.1-clade virus (B.1.319) were used for calculating fold changes in IC50 for variants relative to the 'wild type' (n=15). All donors in this second cohort were included for analysis as they all had positive serum neutralization titres against 'widtype' B.1-clade virus (B.1.319). In cohort 1 and in cohort 2, case sample size was determined to be sufficient for statistical testing. Five IVIG lots (Poly IgG 1033, 4850, 7450, 0301, 0723) manufactured using the Privigen process included US plasma collected by plasmapheresis from a mixture of vaccinated with SARS-CoV-2 mRNA vaccines, convalescent and non-convalescent donors (source plasma, n between 9495-23,667 per batch) with majority of donations collected between April and June 2021. The sample size for nasopharyngeal swabs was dictated by availability of swab specimens from community donors. For the Avalon cluster swabs from 17 donors were tested while for the Delta cluster 80 swab specimens were collected and tested. For Omicron studies, 35 nasopharyngeal swab samples were analysed in this study.
Data exclusions	In the convalescent cohort (ADAPT) 10 donors that did not reach titre with any of the variants except the ancestral strain were excluded while calculating fold changes in neutralization titres (Figure 4 in the manuscript) as it was not possible to calculate IC50 values for these samples.
Replication	Each experiment was run at least thrice and samples were run in quadruplicates. All repeat experiments were successful as observed with appropriate positive and negative controls.
Randomization	Samples from the ADAPT cohort were selected on the basis of serum neutralization titres against 'widtype' B.1-clade virus. As the samples were primarily used for assay cross-validation, it was important to determine neutralisation titers that covered a range of potency. For other samples, they were allocated randomly (vaccine samples and postive nasopharygeal swabs).
Blinding	Investigators were blinded to samples throughout. The only data available for the samples was within the ADAPT cohort, where neutralisation titers were determined from a larger donor pool. From this pool a continuum of responses was assembled as outlined above.

Reporting for specific materials, systems and methods

We require information from authors about some types of materials, experimental systems and methods used in many studies. Here, indicate whether each material, system or method listed is relevant to your study. If you are not sure if a list item applies to your research, read the appropriate section before selecting a response.

Materials & experimental systems

n/a	Involved in the study	
☐	☒	Antibodies
☐	☒	Eukaryotic cell lines
☒	☐	Palaeontology and archaeology
☒	☐	Animals and other organisms
☐	☒	Human research participants
☒	☐	Clinical data
☒	☐	Dual use research of concern

Methods

n/a	Involved in the study	
☒	☐	ChIP-seq
☒	☐	Flow cytometry
☒	☐	MRI-based neuroimaging

Antibodies

Antibodies used	AB-3467, Sotrovimab, Casirivimab, Imdevimab, Bamlanivimab, Cilgavimab and Tixagevimab
Validation	AB-3467 has been extensively characterised in Burnett et al (DOI: 10.1016/j.immuni.2021.10.019).<br>Sotrovimab, Casirivimab, Imdevimab, Bamlanivimab, Cilgavimab and Tixagevimab were generated by gene synthesis, cloned into human IgG1 expression vectors, and produced in ExpiCHO cells.<br>After production in ExpiCHO cells, monoclonal antibodies were characterized for binding to recombinant RBD by biolayer-interferometry (BLI) and for neutralization of live early clade (A2.2) SARS-CoV-2 virus

Eukaryotic cell lines

Policy information about cell lines

Cell line source(s)	Hek 293T cells (Thermofisher Scientific, #R70007)<br>VeroE6-TMPRSS2 (CellBank Australia, JCRB1819)<br>VeroE6 cells (ATCC® CRL-1586™)<br>Primary bronchial epithelial cells (pBEC) from P. A. B. Wark (University of Newcastle)<br>ExpiCHO cells (Thermofisher, A29133)
Authentication	The Garvan Molecular Genetics facility at the Garvan Institute of Medical Research performed cell line authentication on all human cell lines used. DNA from each cell line was analysed for short tandem repeat loci using the PowerPlexR 18D System. All human cell lines listed above were >80% identical, indicating they originated from the cell line specified.
Mycoplasma contamination	All cell lines were tested at the Mycoplasma Testing Facility at UNSW using the Lonza MycoAlertTM Mycoplasma Detection Kit (catalogue number LT07-318). All cell lines used in this study were negative for mycoplasma contamination.
Commonly misidentified lines<br>(See ICLAC register)	No misidentified cell lines were used in this study.

Human research participants

Policy information about studies involving human research participants

Population characteristics	For ADAPT cohort, this has been described in detail in Tea et al (DOI: 10.1371/journal.pmed.1003656). For the samples used in each setting, we have stated median age, interquartile age range and also the female to male ratio. This is outlined in the supplementary tables.<br>Vaccinated donors were drawn from laboratory and healthcare workers, as they were the first groups in Australia to be vaccinated. For the samples used in each setting, we have stated median age, interquartile age range and also the female to male ratio. This is outlined in the supplementary tables.<br>US plasma donor numbers in Polyclonal IgG batches are outlined in the supplementary figures. The median age and female:male ratio is not known.
Recruitment	Convalescent patients from ADAPT were diagnosed at a community-based fever clinic and recruited as outlined by Tea et al (DOI: 10.1371/journal.pmed.1003656). The donors tested herein were assigned based on initial neutralisation titers to the B.1-clade (B.1.319) virus. Donors with high neutralisation titers to early clade variants are biased to being male with a median age of 57. Vaccine donors derived from laboratory and healthcare workers are approximately 2:1 female to male in ratio with a median age of 38.
Ethics oversight	All human serum samples were obtained with written informed consent from the participants and was approved by the St Vincent's Hospital Ethics Committee (2020/ETH00964; 2018/ETH00145; 2021/ETH00180). The use of remnant diagnostic swabs for assessment and development of diagnostic tests, for determining viral genotype, and for in vitro and in vivo research assessing phenotypes such as its determinants of transmissibility, pathogenicity and response to preventive and treatment interventions was under the approval of the NSW CHO following independent scientific review 2021/NSWCHO H21/126831).

Note that full information on the approval of the study protocol must also be provided in the manuscript.
