# Supplementary figures and images for: Platform for isolation and characterization of SARS-CoV-2 variants enables rapid characterization of Omicron in Australia
Source: Nat Microbiol. 2022 May 30;7(6):896–908. doi: 10.1038/s41564-022-01135-7 (PMC9159941; doi:10.1038/s41564-022-01135-7)

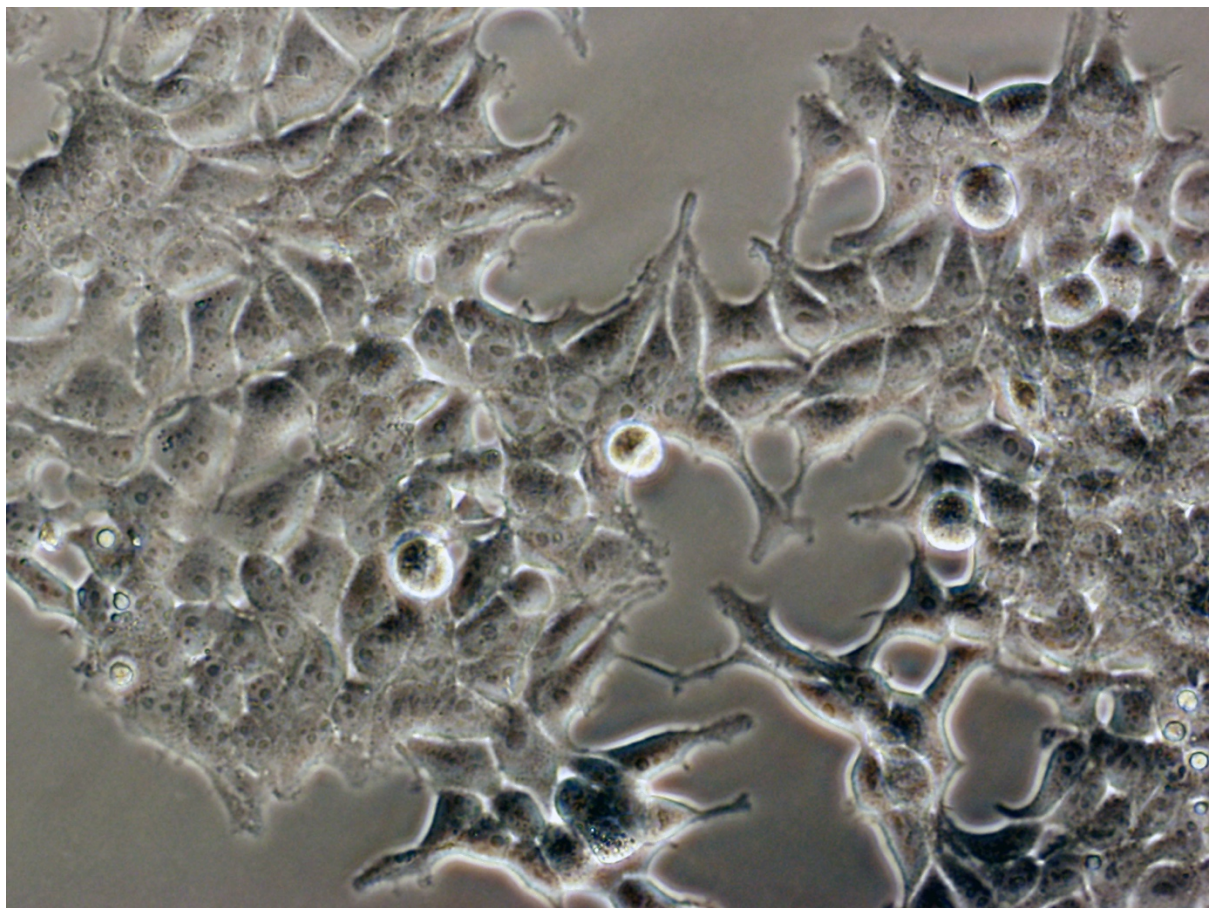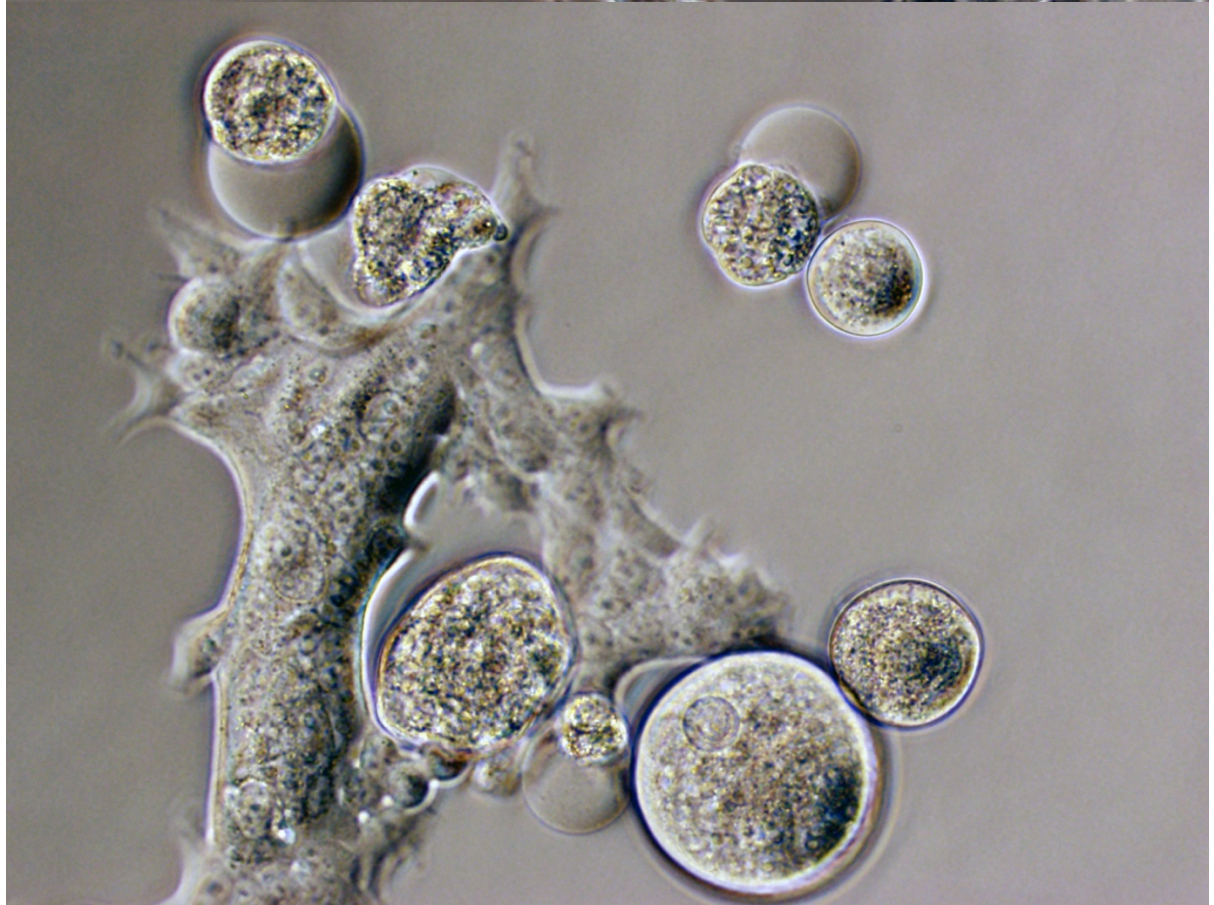

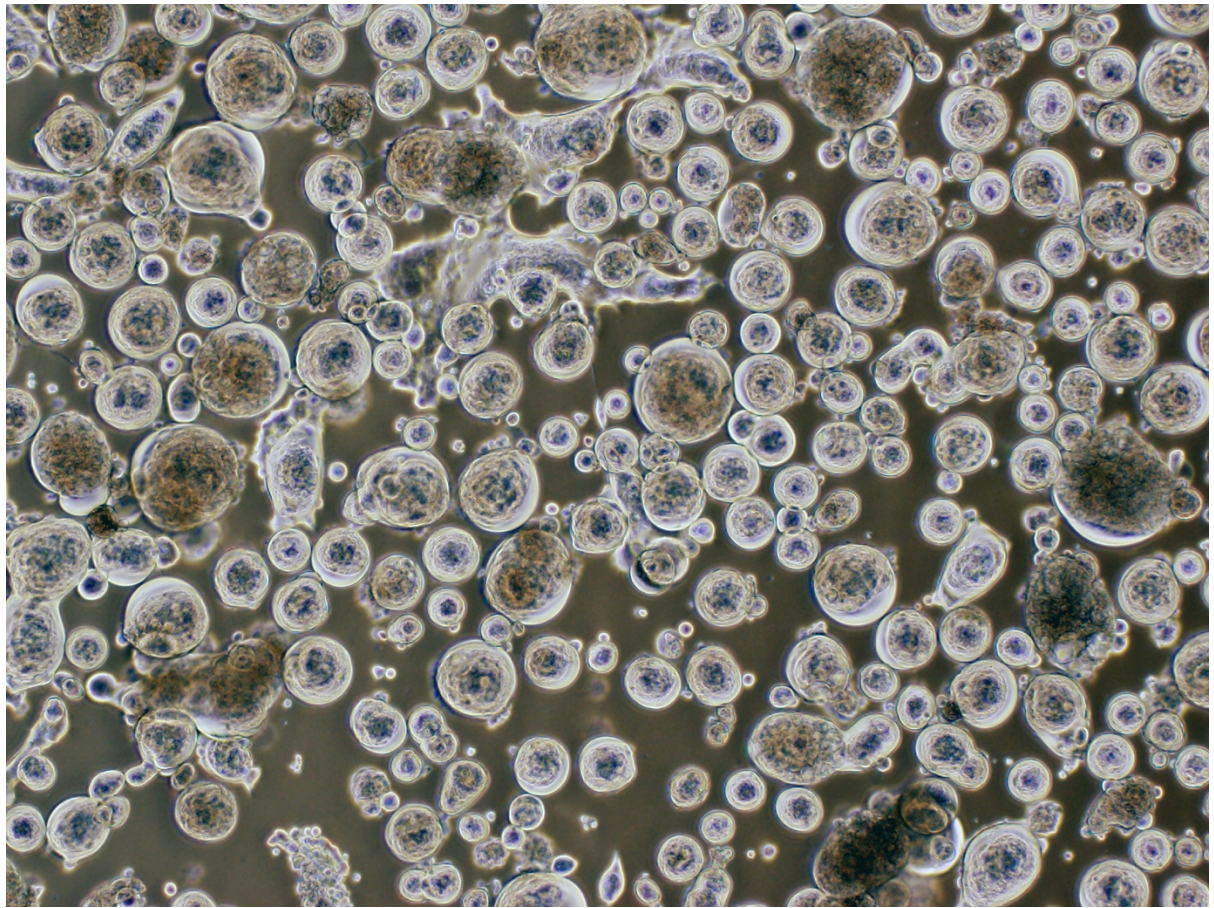

Supplement: Source Data Fig. 1 — Tiff images for Fig. 1. [file 41564_2022_1135_MOESM7_ESM.pdf]

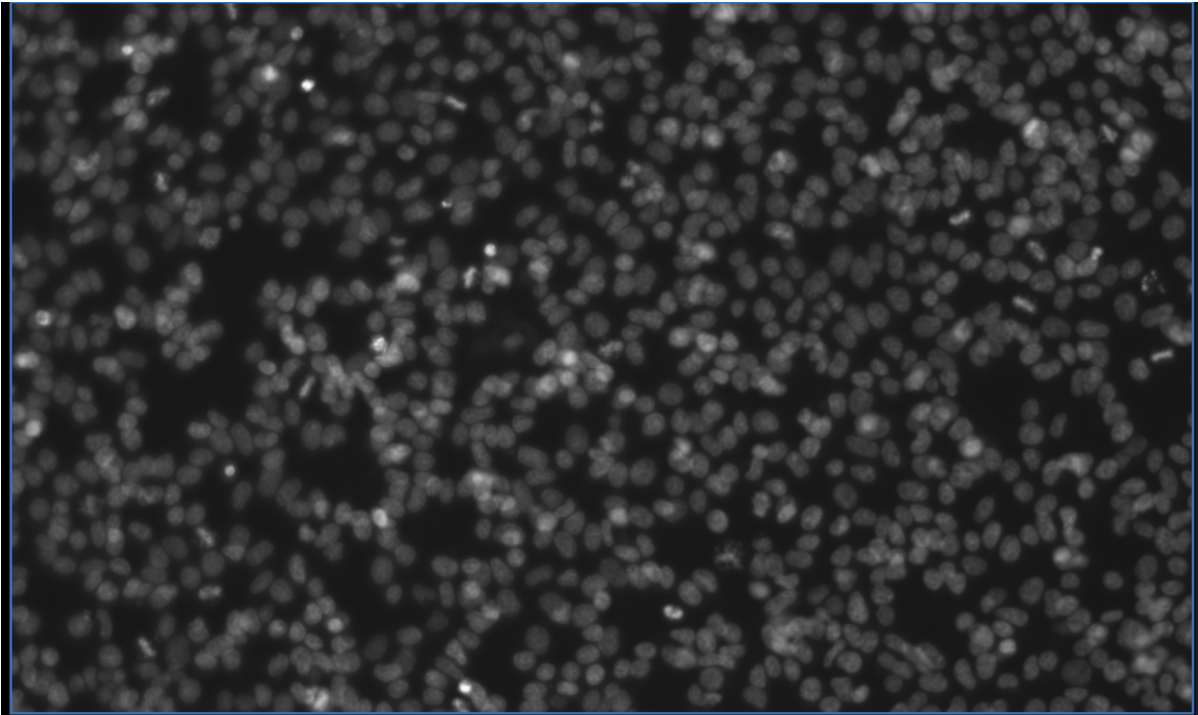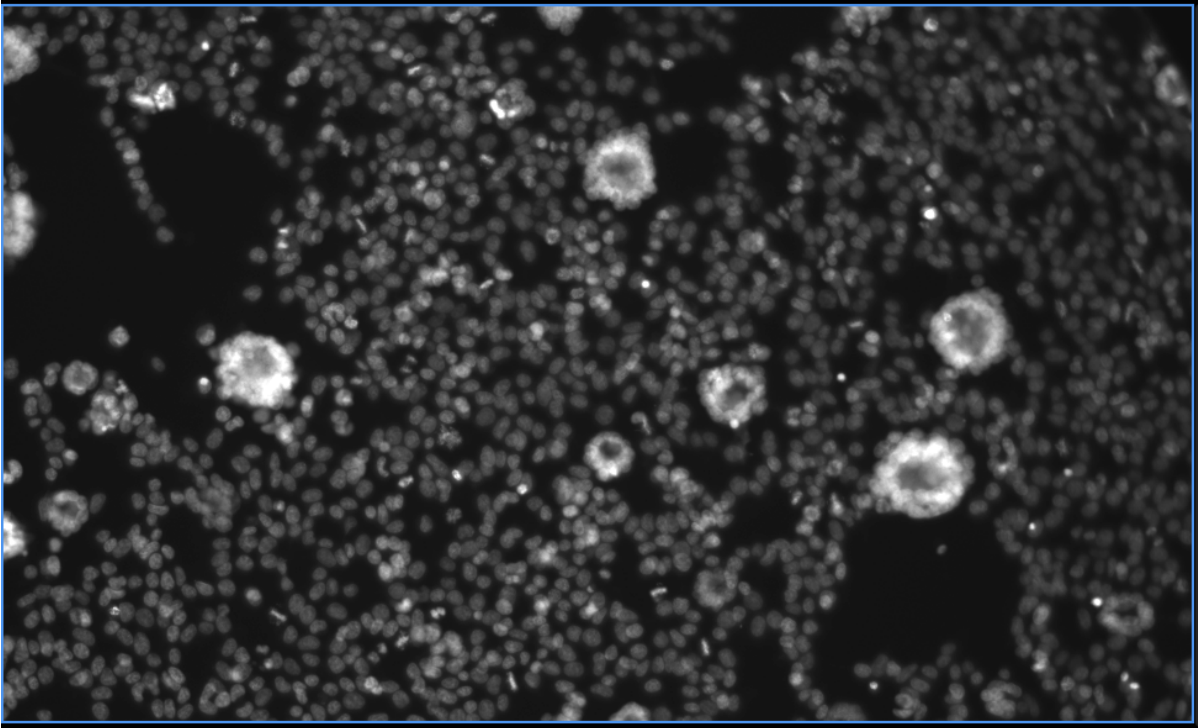

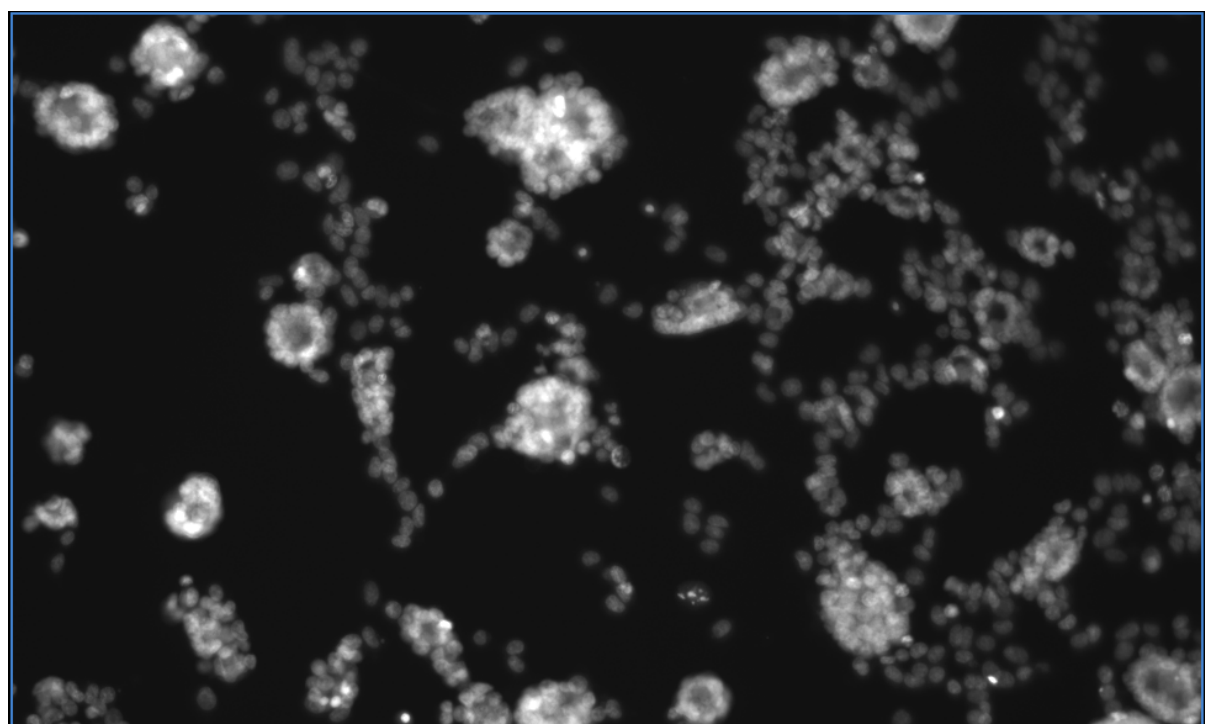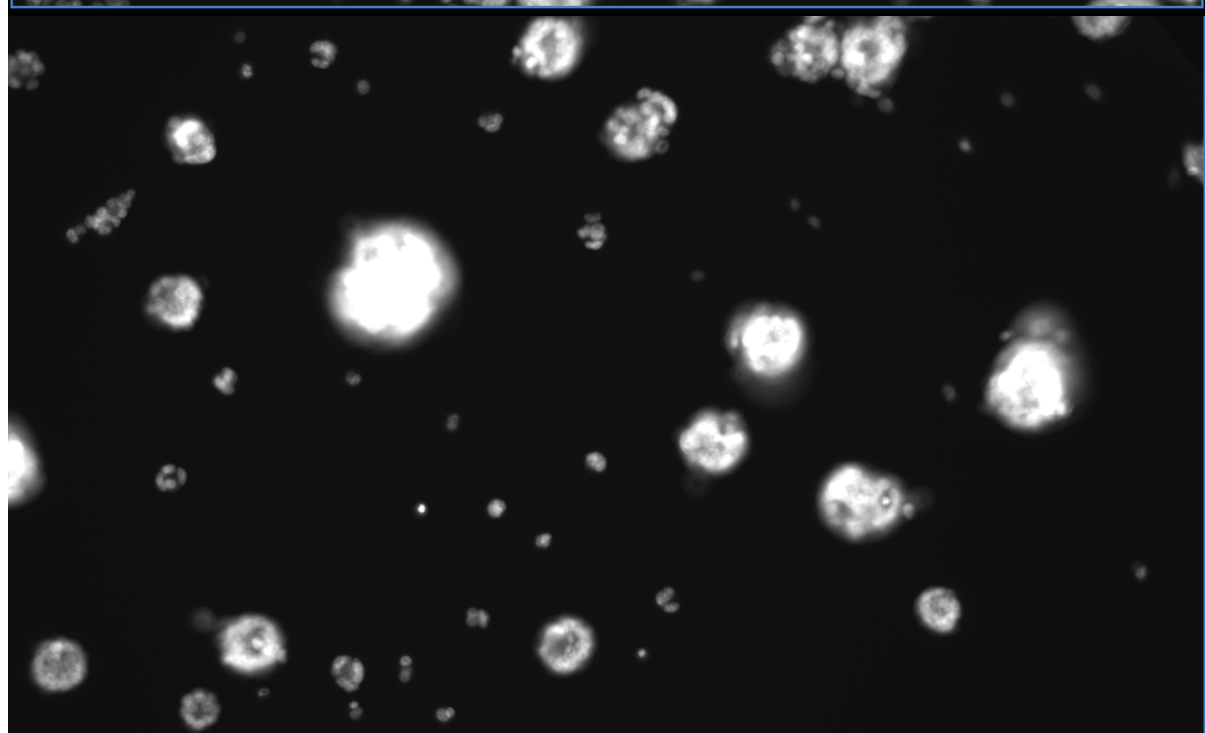

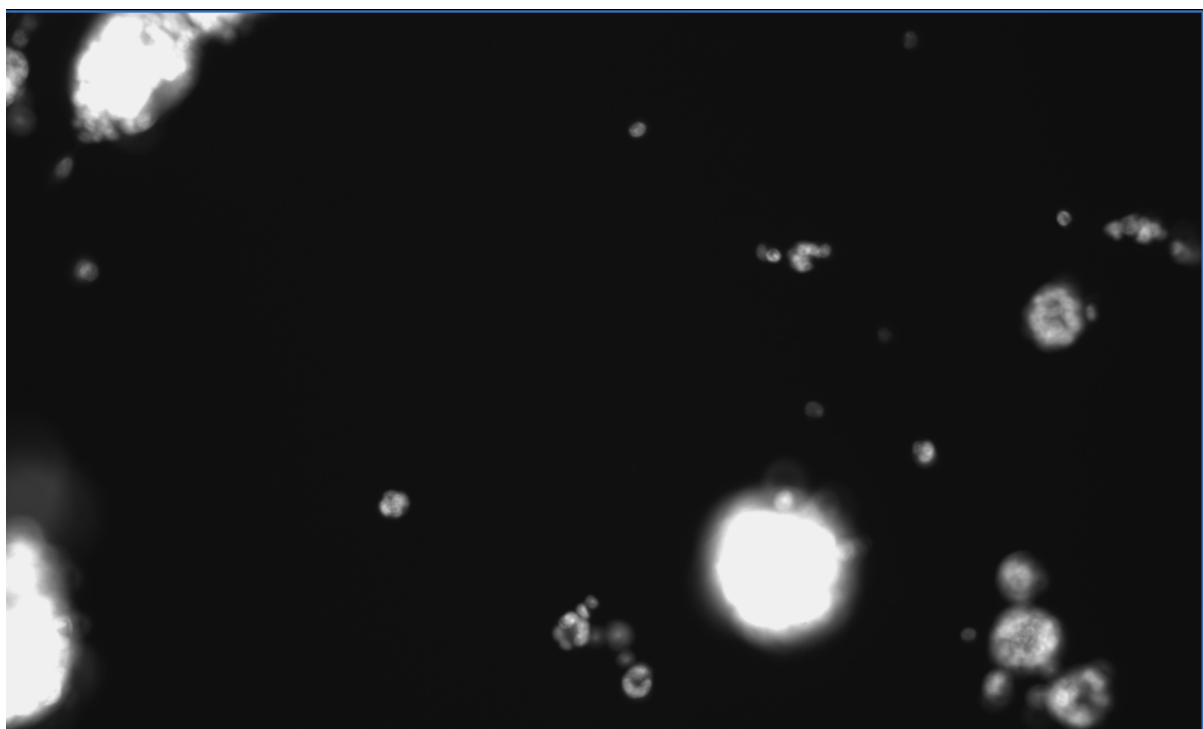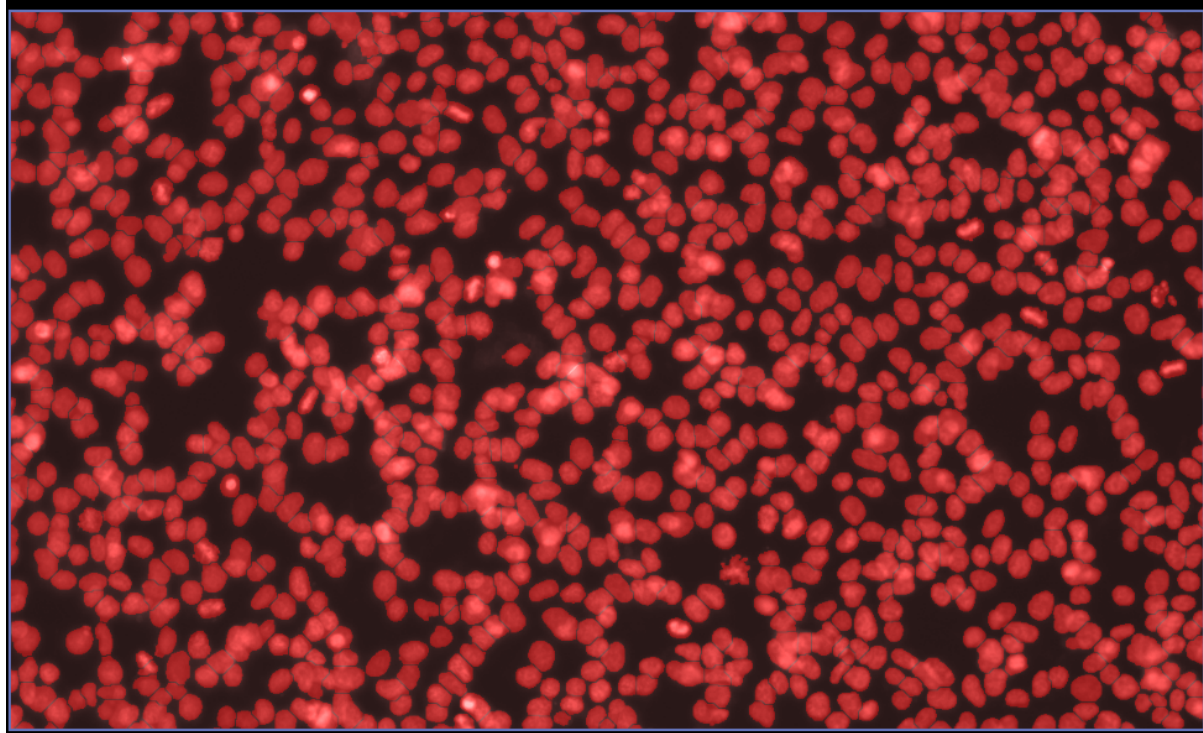

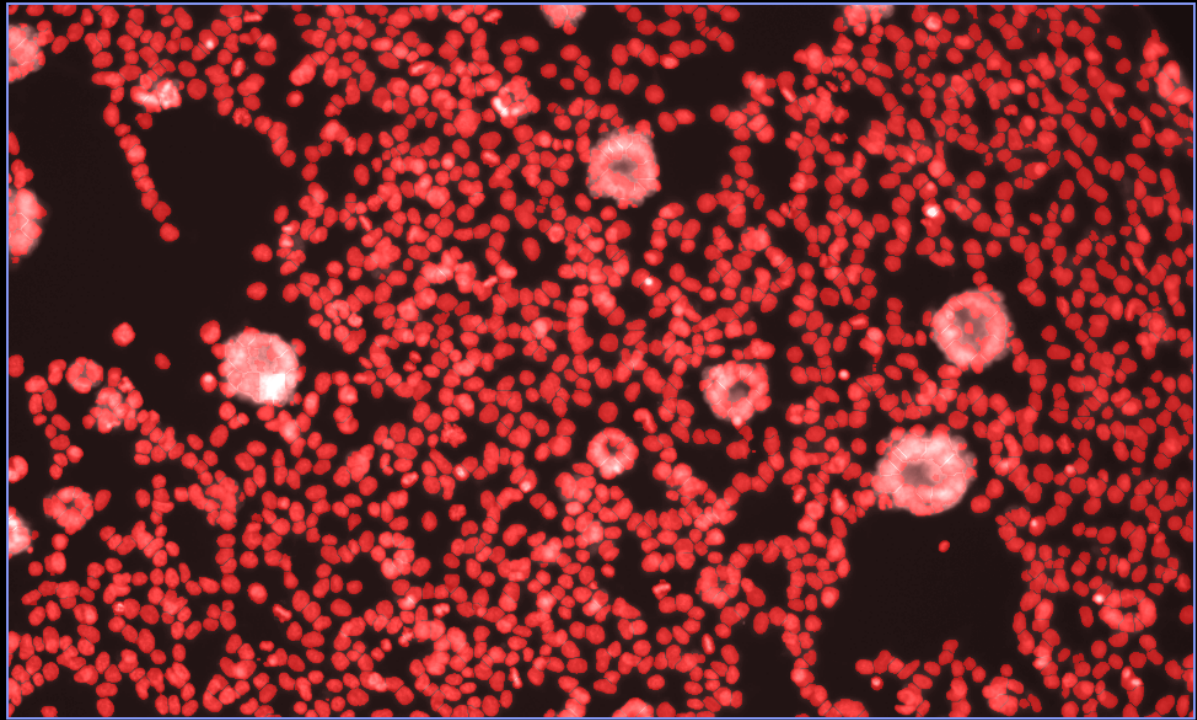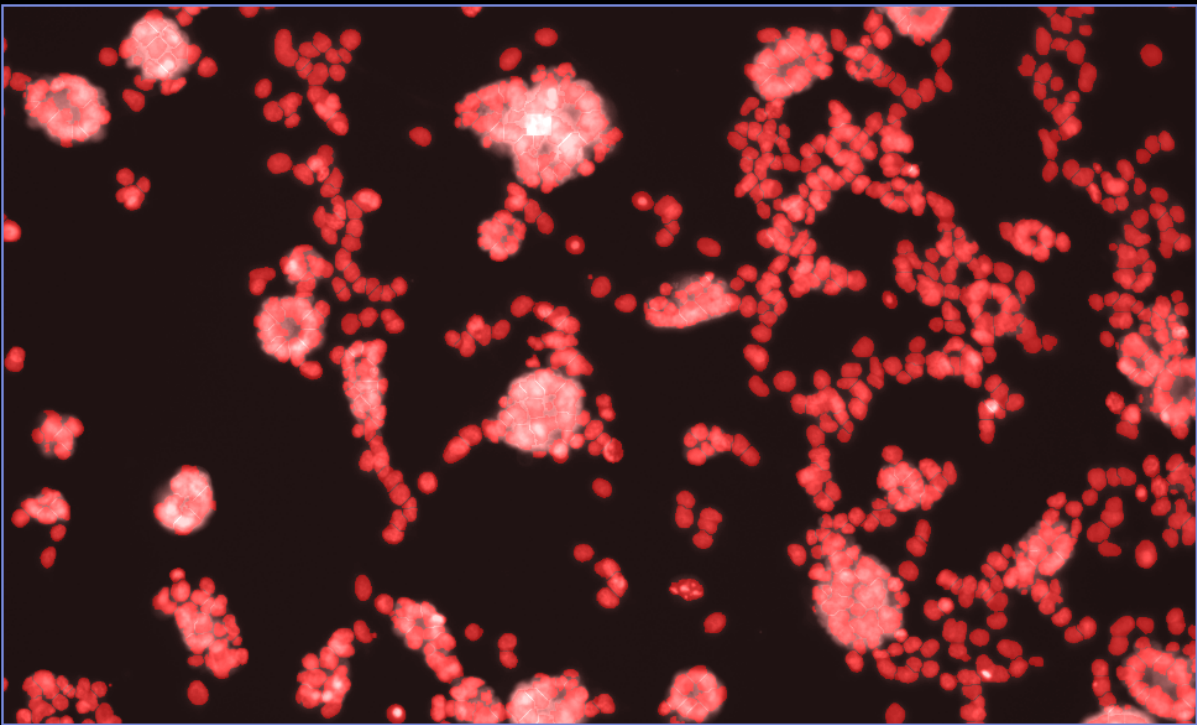

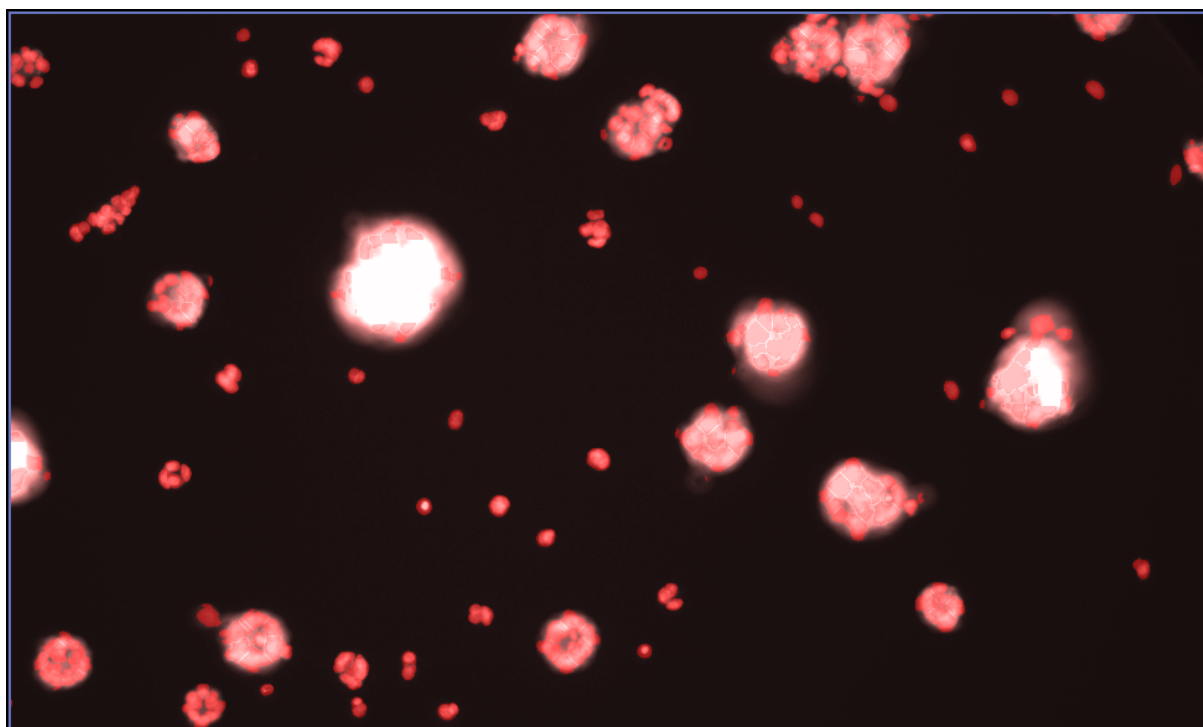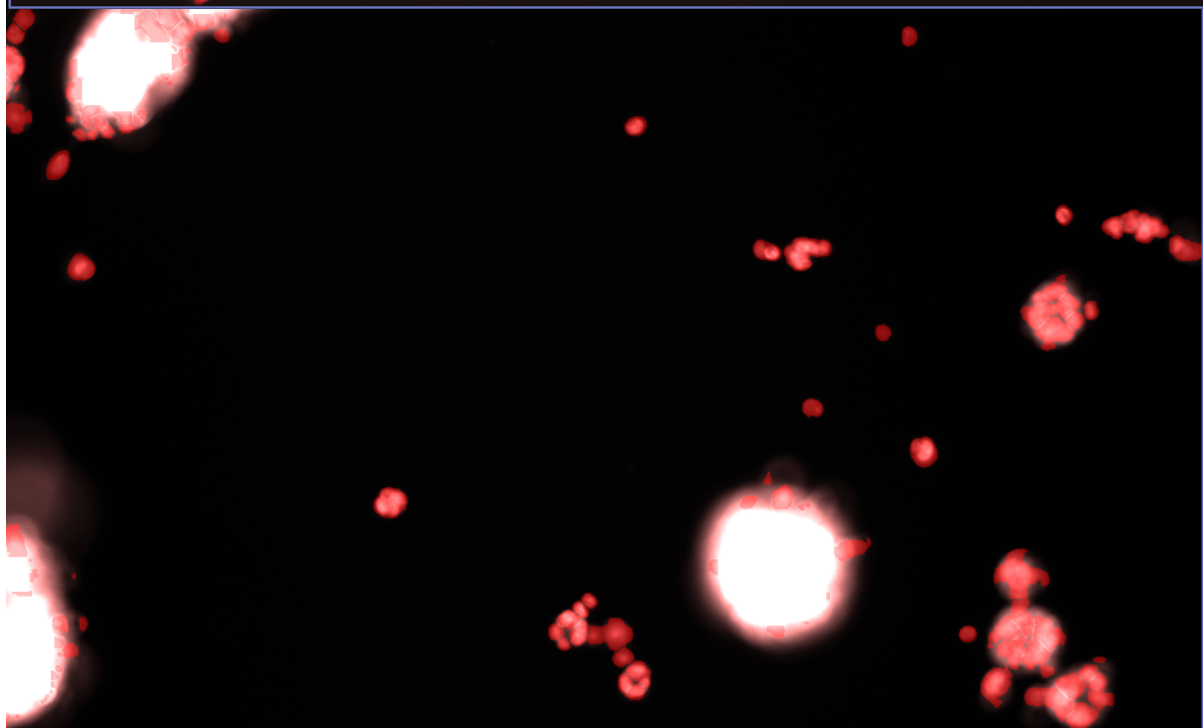

Supplement: Source Data Fig. 2 — Tiff images for Fig. 2. [file 41564_2022_1135_MOESM9_ESM.pdf]

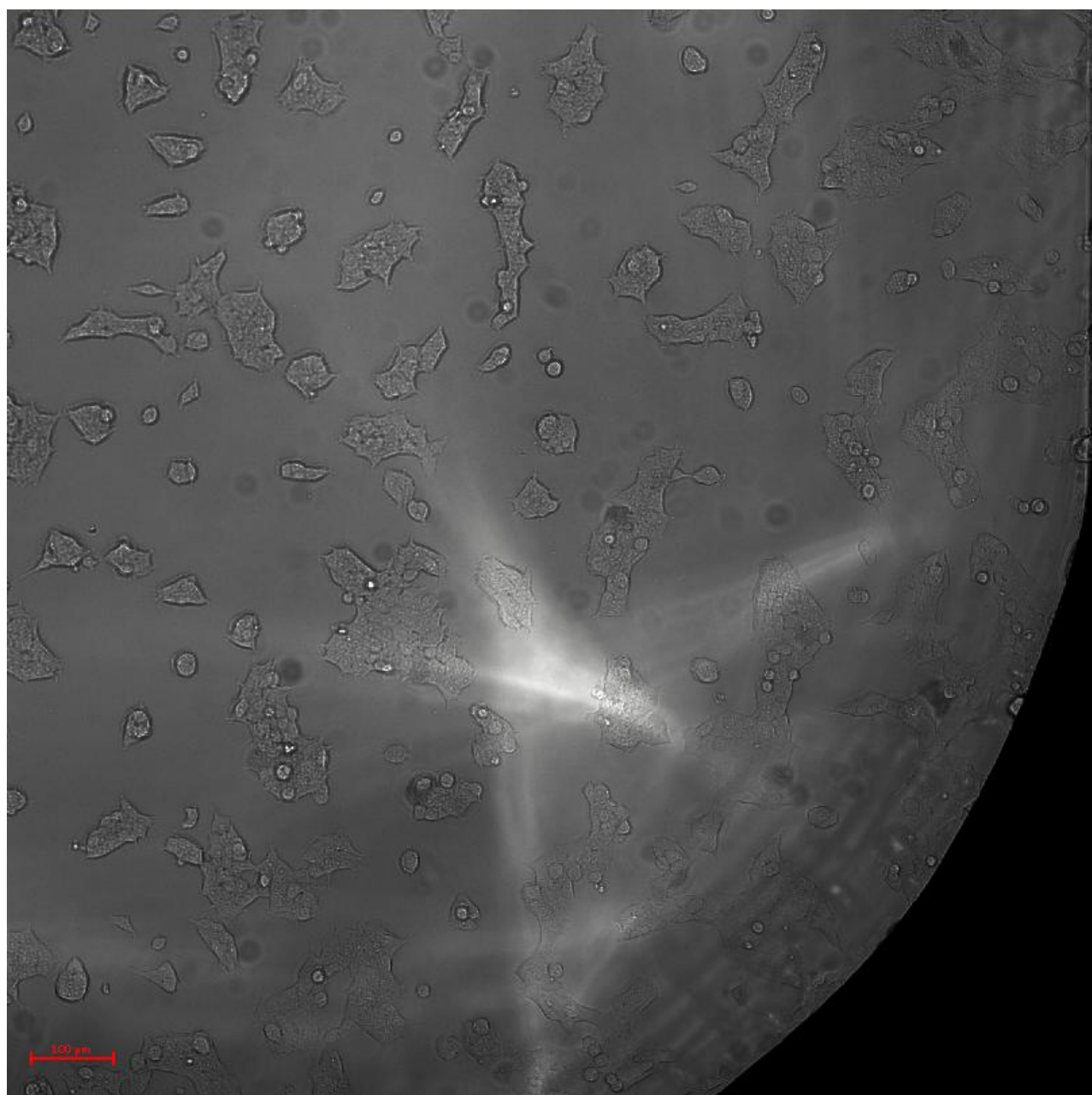

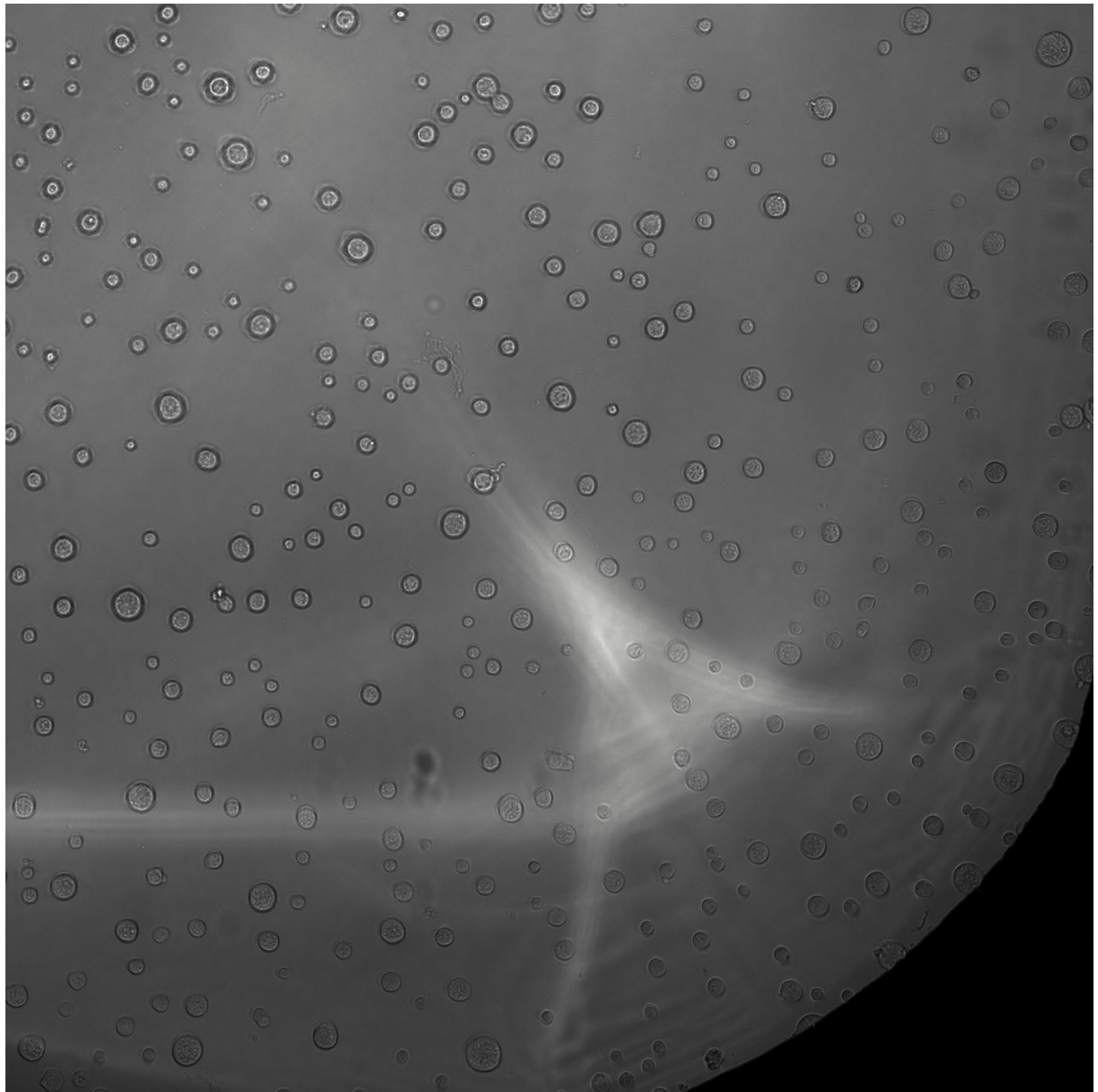

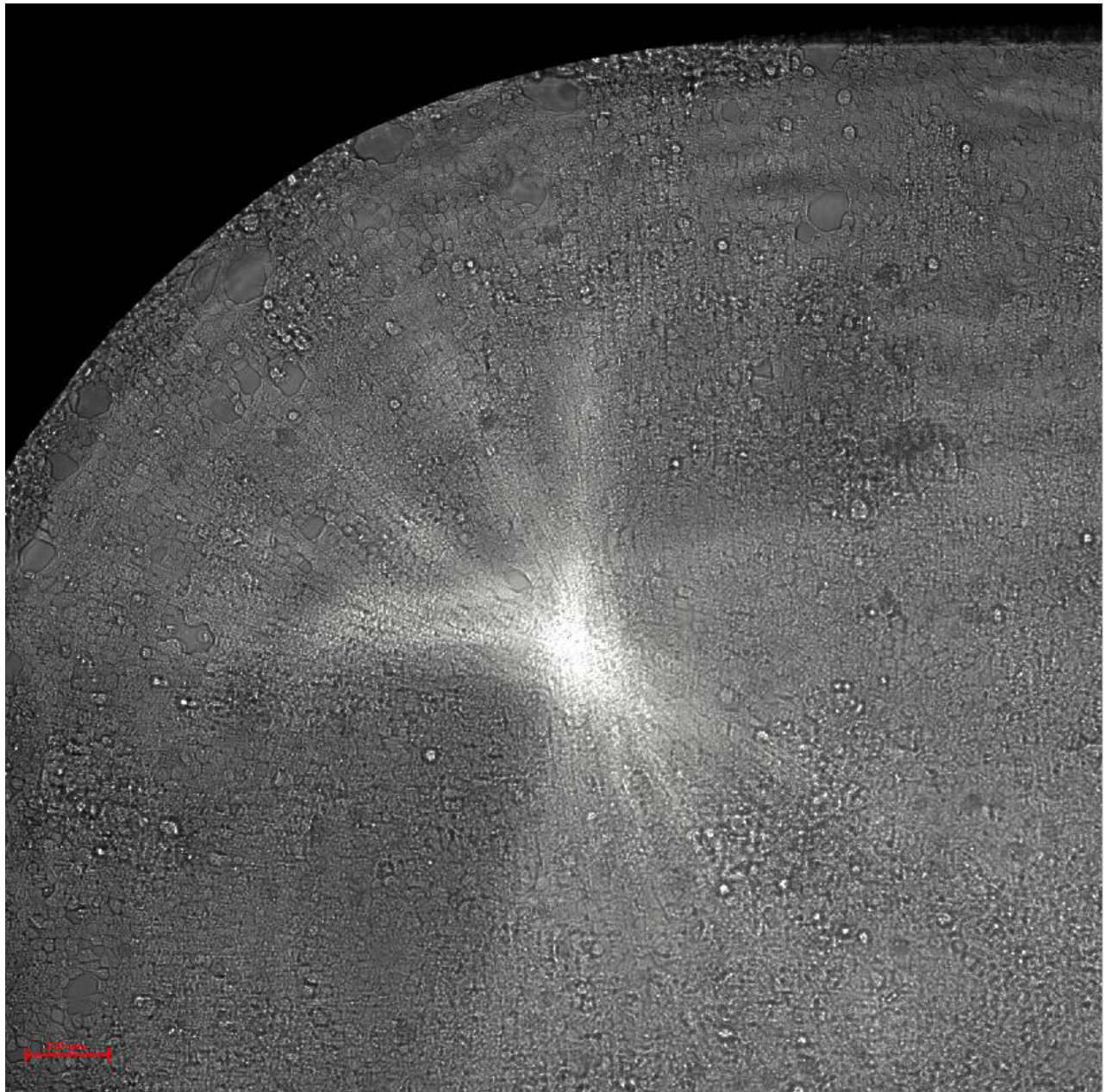

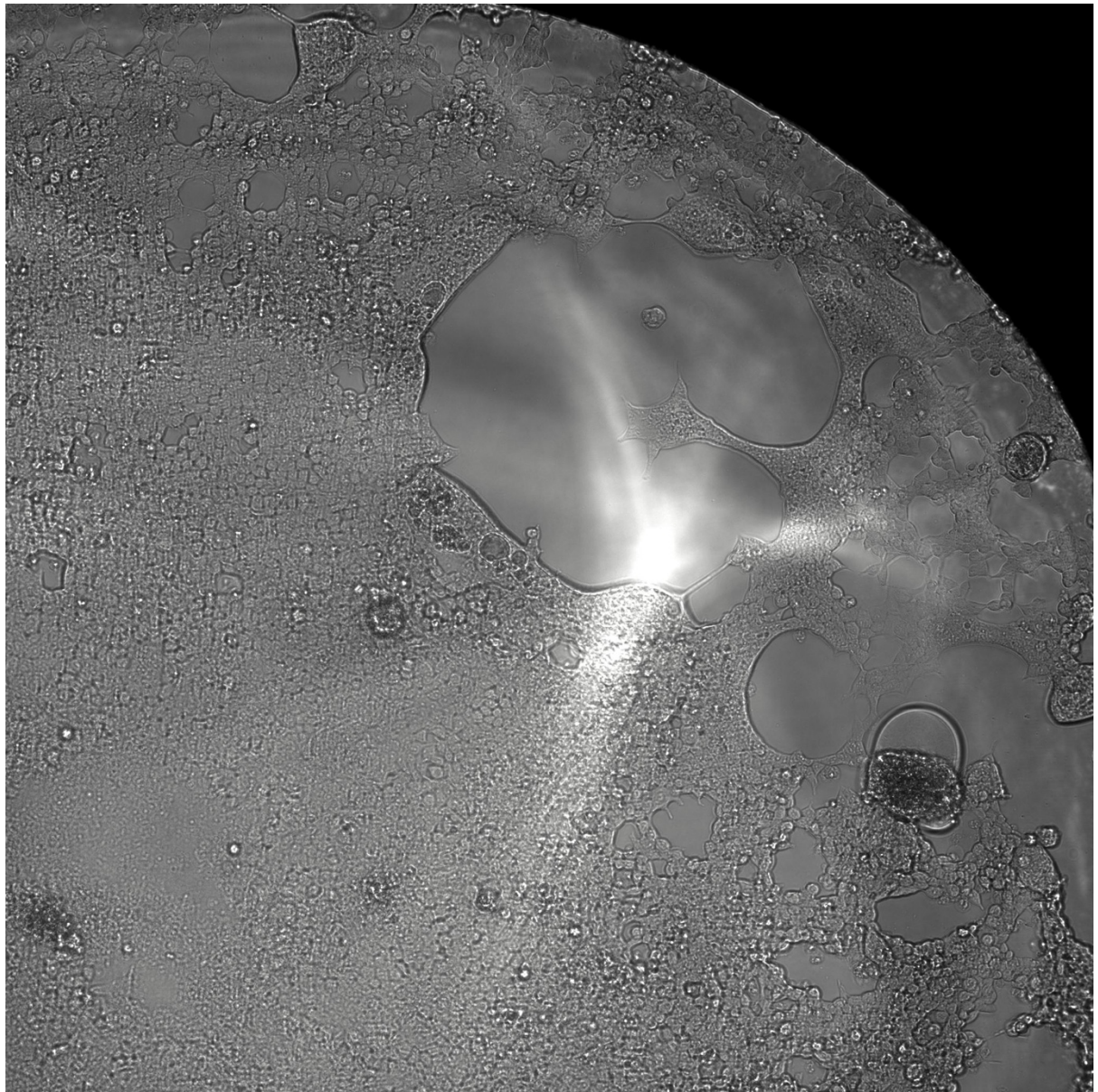

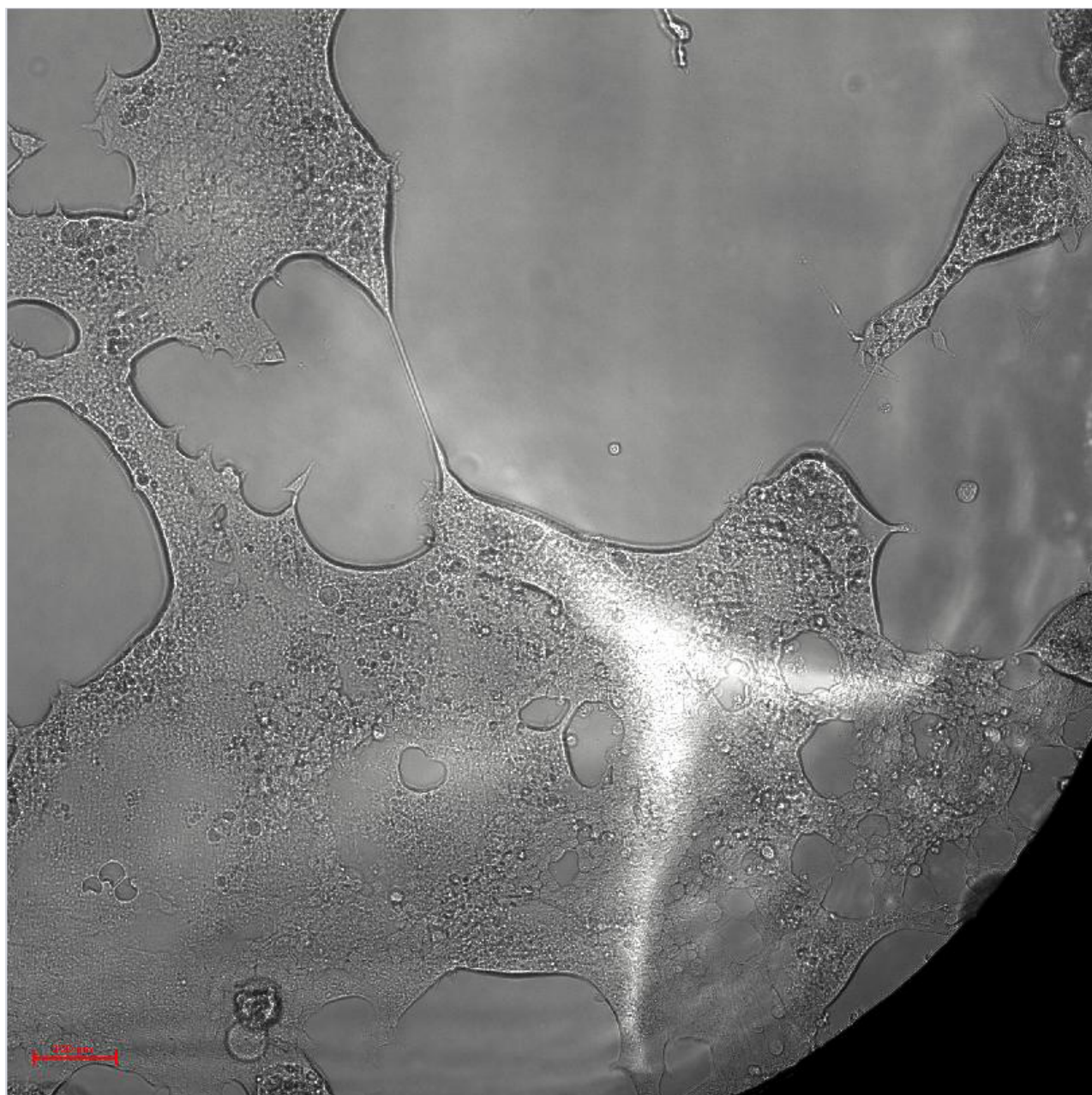

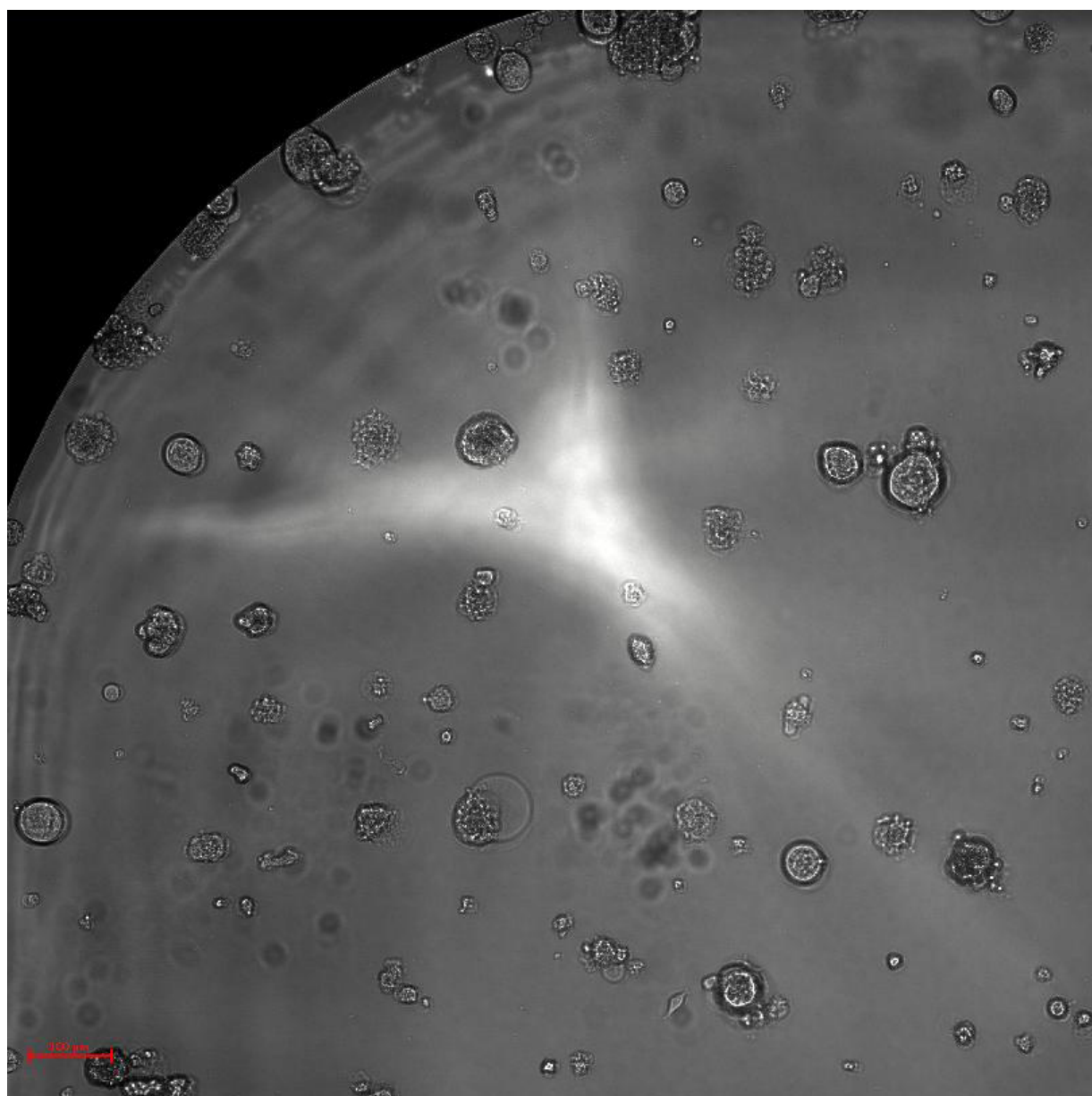

Supplement: Source Data Fig. 4 — Tiff images for Fig. 4. [file 41564_2022_1135_MOESM12_ESM.pdf]

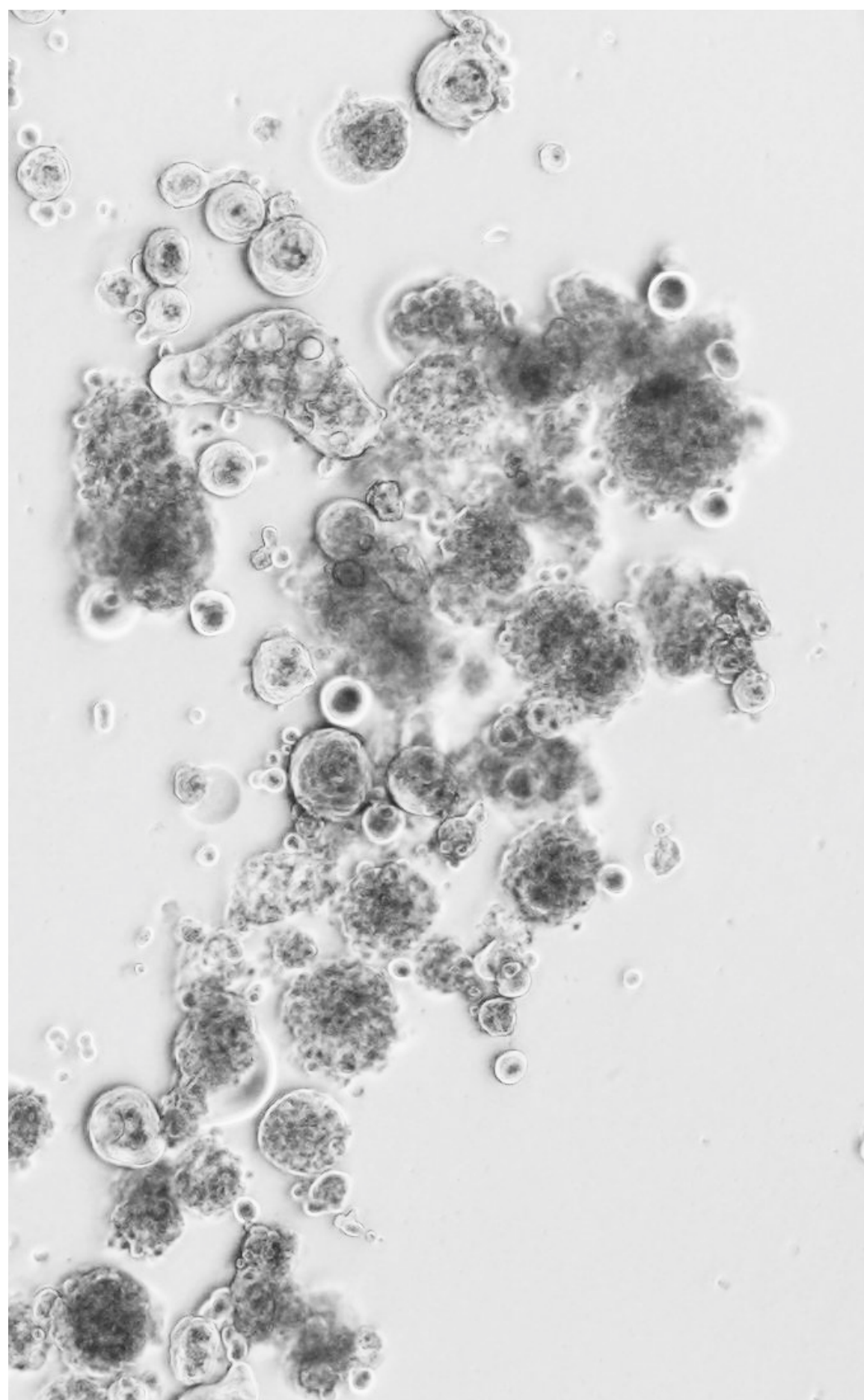

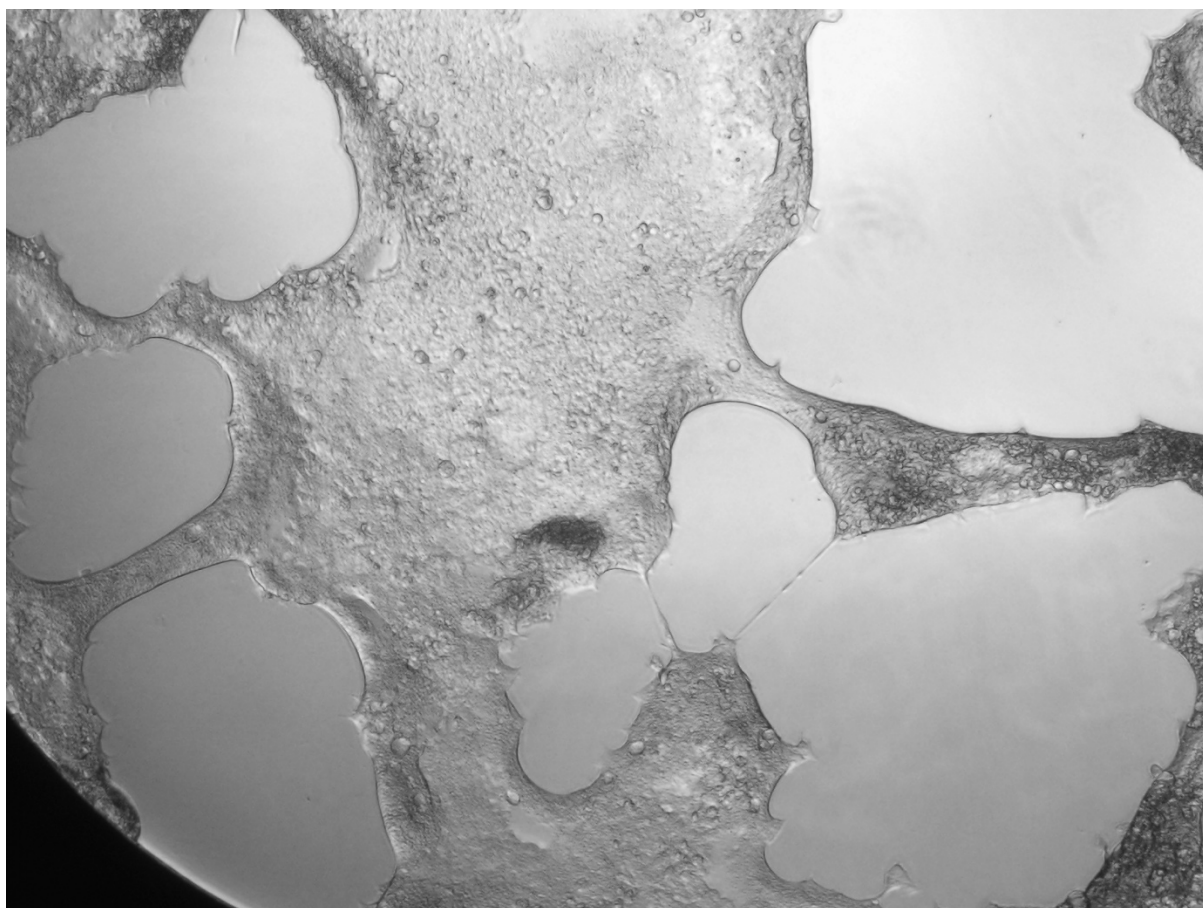

Supplement: Source Data Fig. 6 — Tiff images for Fig. 6. [file 41564_2022_1135_MOESM15_ESM.pdf]
